# Major Adverse Kidney Events in Hospitalized Older Patients With Acute Kidney Injury: Machine Learning–Based Model Development and Validation Study

**DOI:** 10.2196/52786

**Published:** 2025-01-03

**Authors:** Xiao-Qin Luo, Ning-Ya Zhang, Ying-Hao Deng, Hong-Shen Wang, Yi-Xin Kang, Shao-Bin Duan

**Affiliations:** 1 Department of Geriatrics The Second Xiangya Hospital of Central South University Changsha China; 2 Information Center The Second Xiangya Hospital of Central South University Changsha China; 3 Health Management Center National Clinical Research Center for Metabolic Diseases, Hunan Provincial Clinical Medicine Research Center for Intelligent Management of Chronic Disease The Second Xiangya Hospital of Central South University Changsha China; 4 Department of Critical Care Medicine The Second Xiangya Hospital of Central South University Changsha China; 5 Department of Nephrology Hunan Key Laboratory of Kidney Disease and Blood Purification The Second Xiangya Hospital of Central South University Changsha China

**Keywords:** major adverse kidney events within 30 days, older, acute kidney injury, machine learning, prediction model

## Abstract

**Background:**

Acute kidney injury (AKI) is a common complication in hospitalized older patients, associated with increased morbidity, mortality, and health care costs. Major adverse kidney events within 30 days (MAKE30), a composite of death, new renal replacement therapy, or persistent renal dysfunction, has been recommended as a patient-centered endpoint for clinical trials involving AKI.

**Objective:**

This study aimed to develop and validate a machine learning–based model to predict MAKE30 in hospitalized older patients with AKI.

**Methods:**

A total of 4266 older patients (aged ≥ 65 years) with AKI admitted to the Second Xiangya Hospital of Central South University from January 1, 2015, to December 31, 2020, were included and randomly divided into a training set and an internal test set in a ratio of 7:3. An additional cohort of 11,864 eligible patients from the Medical Information Mart for Intensive Care Ⅳ database served as an external test set. The Boruta algorithm was used to select the most important predictor variables from 53 candidate variables. The eXtreme Gradient Boosting algorithm was applied to establish a prediction model for MAKE30. Model discrimination was evaluated by the area under the receiver operating characteristic curve (AUROC). The SHapley Additive exPlanations method was used to interpret model predictions.

**Results:**

The overall incidence of MAKE30 in the 2 study cohorts was 28.3% (95% CI 26.9%-29.7%) and 26.7% (95% CI 25.9%-27.5%), respectively. The prediction model for MAKE30 exhibited adequate predictive performance, with an AUROC of 0.868 (95% CI 0.852-0.881) in the training set and 0.823 (95% CI 0.798-0.846) in the internal test set. Its simplified version achieved an AUROC of 0.744 (95% CI 0.735-0.754) in the external test set. The SHapley Additive exPlanations method showed that the use of vasopressors, mechanical ventilation, blood urea nitrogen level, red blood cell distribution width-coefficient of variation, and serum albumin level were closely associated with MAKE30.

**Conclusions:**

An interpretable eXtreme Gradient Boosting model was developed and validated to predict MAKE30, which provides opportunities for risk stratification, clinical decision-making, and the conduct of clinical trials involving AKI.

**Trial Registration:**

Chinese Clinical Trial Registry ChiCTR2200061610; https://tinyurl.com/3smf9nuw

## Introduction

Acute kidney injury (AKI) is a clinical syndrome characterized by a rapid decline in renal function [1]. The incidence of AKI has been reported to be about 10%-15% in hospitalized patients and over 50% in critically ill patients [2-4]. AKI is common in older individuals, associated with increased morbidity, mortality, and health care costs [5-7]. The prevalence of multiple comorbidities as well as age-related changes in the kidneys, systemic vasculature, and immune system render older patients more susceptible to kidney damage and less likely to recover [5,8]. Given the already high and increasing incidence of AKI in the older, there is a tremendous need to develop clinical tools for risk stratification of postAKI outcomes in this age group.

Major adverse kidney events within 30 days (MAKE30), a composite of death, new renal replacement therapy (RRT), or persistent renal dysfunction (PRD), has been recognized as a key metric of post-AKI outcomes [9,10]. Patients with no renal recovery during hospitalization show a higher risk of long-term adverse outcomes than those who recovered [11,12]. Furthermore, the initiation of RRT after AKI onset is associated with long-term renal dysfunction and death [13,14]. Previous studies have confirmed MAKE30 as a common, feasible to measure, and clinically meaningful endpoint in clinical trials involving AKI [15-17]. The prediction of MAKE30 can be critical for evaluating patient prognosis, guiding clinical decision-making, and facilitating the conduct of clinical trials.

Clinical prediction models are mathematical tools that are primarily intended to assist physicians in their clinical decision-making [18]. Recently, the rapid development in medical big data and advances in computer science have sparked a growing interest in applying machine learning techniques to develop clinical prediction models [19]. Compared with traditional statistical methods, advanced machine learning algorithms can better integrate large amounts of clinical data, fit complex nonlinear relationships, and analyze high-order interactions. The eXtreme Gradient Boosting (XGBoost) algorithm, an efficient implementation of the gradient boosting framework, represents one of the notable advances in machine learning and is widely used in the medical field [20-23]. XGBoost excels in preventing overfitting during the training process, enhancing predictive performance and robustness in complex data scenarios.

Therefore, the objective of this study was to develop and validate a clinical prediction model for MAKE30 in hospitalized older patients with AKI using the machine learning XGBoost algorithm.

## Methods

### Study Design

This study identified older patients (aged ≥ 65 years) with AKI who were admitted to the Second Xiangya Hospital of Central South University from January 1, 2015, to December 31, 2020. AKI was defined according to the Kidney Disease: Improving Global Outcomes criteria as an increase in serum creatinine (SCr) by ≥26.5 μmol/L within 48 hours or to ≥1.5 times baseline within 7 days [24]. Baseline SCr was defined as the lowest creatinine in the past 7 days. AKI was determined every time a SCr measurement occurred based on the changes in SCr over the past 48 hours and 7 days. Patients were excluded if they were diagnosed with end-stage renal disease, had a hospital stay of less than 48 hours, had an initial SCr≥353.6 μmol/L at admission, or required RRT before the diagnosis of AKI.

In addition, a cohort containing eligible patients from the Medical Information Mart for Intensive Care Ⅳ (MIMIC-Ⅳ) database was used for model external validation [25]. MIMIC-Ⅳ is a relational database containing comprehensive information for intensive care unit (ICU) admissions at the Beth Israel Deaconess Medical Center (BIDMC) from 2008 to 2019.

### Data Collection

Data on demographics, diagnoses, laboratory tests, interventions, and medications were collected. A total of 53 candidate predictor variables were identified that were considered clinically relevant to MAKE30, were available in the electronic health records (EHRs), and had a proportion of missing values less than 30% (Multimedia Appendix 1). Comorbidities were determined based on diagnoses encoded in the *ICD-9* (*International Classification of Diseases, Ninth Edition*) or *ICD-10* (*International Statistical Classification of Diseases, Tenth Revision*). The burden of comorbidities was assessed by the Charlson comorbidity index [26,27]. Laboratory tests were recorded as the measurements within 48 hours of and closest to the diagnosis of AKI. Medications were identified according to the Anatomic Therapeutic Chemical classification system and included if administered within 48 hours after the diagnosis of AKI [28]. The use of mechanical ventilation within 48 hours after the diagnosis of AKI was also collected.

### Outcome Measures

The primary outcome was MAKE30, defined as a composite of death, new RRT, or PRD at hospital discharge or at 30 days after the diagnosis of AKI, whichever occurred first [9,10]. PRD was defined as a final inpatient SCr value ≥2 times baseline. Secondary outcomes were length of hospital stay as well as death within 30 days and 1 year after discharge. Survival data were extracted from the Chinese Center for Disease Control and Prevention cause-of-death reporting system.

### Statistical Analysis

Statistical analyses were performed using R software (version 4.1.2; R Foundation for Statistical Computing). Categorical data are presented as numbers and percentages and were analyzed with the chi-square test or Fisher exact test, as appropriate. Continuous data are described with median (IQR) and were compared using the Mann-Whitney *U* test. Survival data were analyzed with the Kaplan-Meier method, and differences in survival were evaluated by the log-rank test. The 2-sided α level was set at .05. Missing data were analyzed by the missForest method, which is a nonparametric missing value imputation for mixed-type data [29]. The description of missing data can be found in Multimedia Appendix 2.

Patients from the Second Xiangya Hospital were randomly divided into a training set and an internal test set in a ratio of 7:3. The cohort of patients from the MIMIC-Ⅳ database was used as an external test set.

### Feature Selection

To build a parsimonious model, feature selection was performed on the training set. First, we removed the predictor variables with near-zero variance. Near-zero variance was defined as a situation where a variable exhibits very little variation or almost no variability across its values, characterized by over 95% of its values being identical. Then the Boruta algorithm was used to select the most important predictor variables [30]. Boruta is an all-relevant feature selection wrapper algorithm, capable of working with classification methods that output variable importance measures; in this study, the Random Forest model was used. There were 30 variables ultimately selected for inclusion within the machine learning model (Multimedia Appendix 1).

### Model Development and Validation

A machine learning XGBoost model was established on the training set to predict the development of MAKE30 [31]. XGBoost is an optimized implementation of the gradient boosting framework, which sequentially adds weak models (decision trees) to iteratively improve the overall prediction. XGBoost improves upon traditional gradient boosting algorithms by offering scalability, regularization techniques, optimized tree construction, and customization options. A set of model hyperparameters were optimized by running 5 random shuffles of 5-fold cross-validation, including eta, max_depth, min_child_weight, gamma, colsample_bytree, and subsample. The descriptions and search ranges for the hyperparameters are listed in Multimedia Appendix 3. The XGBoost model can be accessed at [LuoXiaoqin123/MAKE30-in-elderly-AKI] (GitHub).

The performance of the XGBoost model was evaluated on the internal and external test sets. Model discrimination was assessed by the area under the receiver operating characteristic curve (AUROC), which is an assessment metric that remains unaffected by incidence or threshold selection. Model calibration was evaluated using the calibration plot aggregated by deciles and the Brier score. The area under the precision-recall curve (AUPRC) was calculated, considering that it is a useful metric for class-imbalanced data in a problem setting where finding the adverse outcome is crucial. A decision curve analysis was performed to illustrate the clinical use of the model. In addition, we determined the optimal cutoff by the receiver operating characteristic (ROC) curve and the maximum Youden index (sensitivity + specificity – 1) in the training set. The sensitivity, specificity, positive predictive value (PPV), negative predictive value (NPV), positive likelihood ratio (PLR), and negative likelihood ratio (NLR) at the optimal cutoff were calculated.

In the external validation set, there were 9 predictor variables with a proportion of missing values exceeding 50% (Multimedia Appendix 2). In this case, we simplified the model to include 21 variables, with model development performed as described above using the same training set.

### Model Interpretations

We used the SHapley Additive exPlanations (SHAP) method to interpret model predictions [32]. SHAP is a game theoretic approach that helps in explaining the output of any machine learning model. It can provide consistent and locally accurate attribution values, the SHAP values, for each feature within a model. A positive SHAP value represents a higher risk of the outcome, whereas a negative SHAP value represents a lower risk of the outcome. The contribution of a feature to the predicted risk of the outcome can be explained by the cumulative effects of feature attribution in each observation.

### Sensitivity Analyses

In order to mitigate the potential impact of age on our findings, we further conducted sensitivity analyses to assess the model’s robustness across patients stratified by age. We evaluated the model’s performance in distinct age groups: <70 years, 70 to 80 years, and ≥80 years.

### Ethical Considerations

The medical ethics committee of the Second Xiangya Hospital of Central South University approved the study protocol (2022-K031) and waived the requirement for informed consent. This project has been registered in Chinese Clinical Trial Registry (ChiCTR2200061610). The institutional review boards of BIDMC and the Massachusetts Institute of Technology approved the project and waived the requirement for informed consent. The study followed the Declaration of Helsinki and the Transparent Reporting of a multivariable prediction model for Individual Prognosis or Diagnosis statement [33].

## Results

### Patient Characteristics

**Figure 1:**
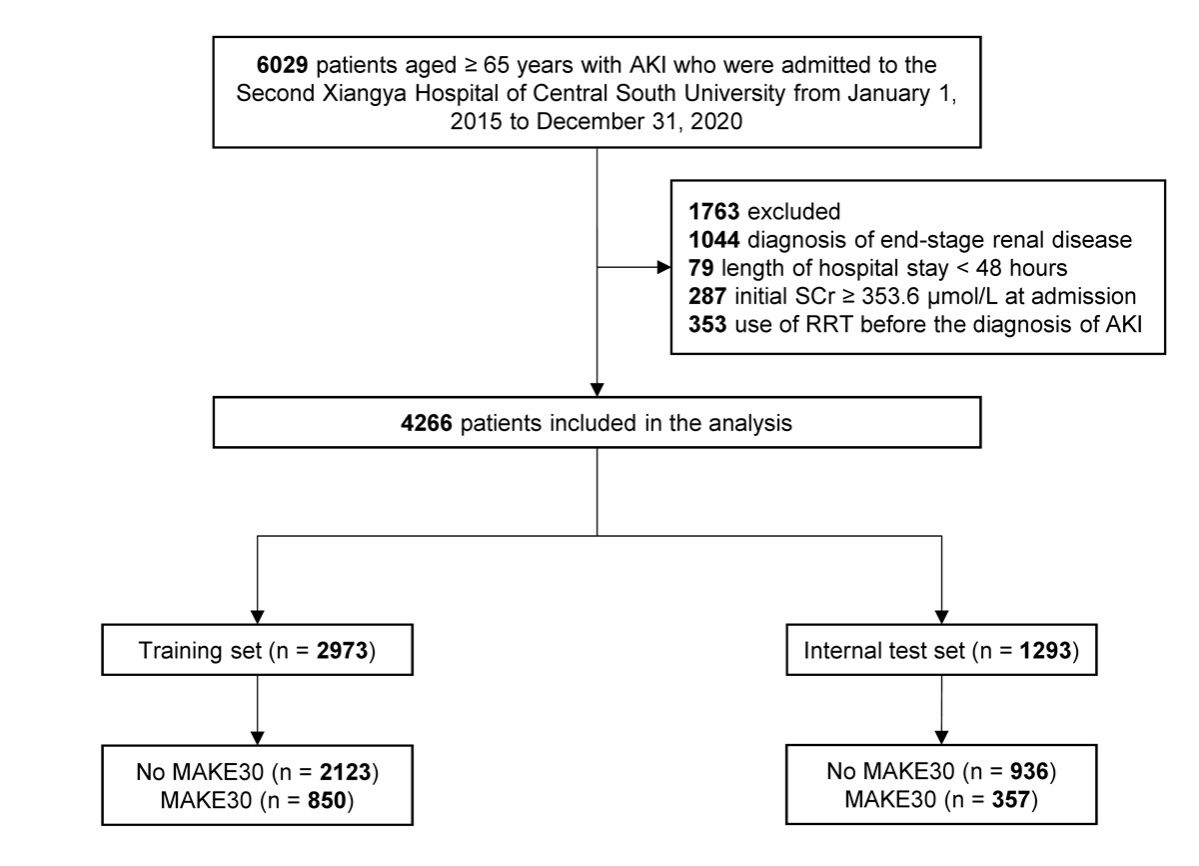
Flow diagram of patient selection from the Second Xiangya Hospital. AKI: acute kidney injury; MAKE30, major adverse kidney events within 30 days; RRT: renal replacement therapy; SCr: serum creatinine.

A total of 4266 patients from the Second Xiangya Hospital were enrolled, including 2973 patients in the training set and 1293 patients in the internal test set (Figure 1). The overall incidence of MAKE30 in this medical center was 28.3% (95% CI 26.9%-29.7%), including 12.3% (95% CI 11.4%-13.4%) with death, 10.2% (95% CI 9.3%-11.1%) with new RRT, and 17.7% (95% CI 16.6%-18.9%) with PRD. The external test set contained 11,864 patients from the MIMIC-Ⅳ database, 26.7% (95% CI 25.9%-27.5%) of whom developed MAKE30 (Multimedia Appendix 4). The incidence of death, new RRT, and PRD were 18.9% (95% CI 18.2%-19.6%), 4.9% (95% CI 4.5%-5.3%), and 12.8% (95% CI 12.2%-13.4%), respectively.

Table 1 shows the characteristics of patients from the Second Xiangya Hospital stratified by MAKE30. Patients with MAKE30 were older, had a higher burden of comorbidities, and had greater disease severity than patients without MAKE30. Compared with the nonMAKE30 group, the MAKE30 group had shorter length of hospital stay as well as higher 30-day and 1-year mortality after discharge. Multimedia Appendix 5 shows the Kaplan-Meier survival curves of death within 30 days and 1 year for patients alive at discharge stratified by PRD or RRT. Patients with versus without new RRT or PRD had higher 30-day and 1-year mortality after discharge (log-rank *P*<.001). Multimedia Appendix 6 provides the characteristics of patients from the MIMIC-Ⅳ database stratified by MAKE30. Multimedia Appendix 7 provides the characteristics of patients in the training, internal test, and external test sets.

**Table 1:** Characteristics of patients from the Second Xiangya Hospital.

Variables			No MAKE30^a^ (n=3059)		MAKE30^a^ (n=1207)		*P* value
Age (years), median (IQR)			72 (68-77)		74 (68-80)		<.001
Sex, male, n (%)			1992 (65.1)		776 (64.3)		.64
Intensive care, n (%)			1284 (42.0)		765 (63.4)		<.001
**Comorbidities, n (%)**							
	Sepsis	127 (4.2)		237 (19.6)		<.001	
	Hypertension	1685 (55.1)		676 (56.0)		.61	
	Myocardial infarction	346 (11.3)		170 (14.1)		.01	
	Congestive heart failure	932 (30.5)		502 (41.6)		<.001	
	Peripheral vascular disease	455 (14.9)		202 (16.7)		.14	
	Cerebrovascular disease	611 (20.0)		347 (28.7)		<.001	
	Dementia	45 (1.5)		41 (3.4)		<.001	
	Chronic pulmonary disease	515 (16.8)		259 (21.5)		<.001	
	Rheumatic disease	60 (2.0)		27 (2.2)		.65	
	Peptic ulcer disease	99 (3.2)		59 (4.9)		.01	
	Liver disease	630 (20.6)		308 (25.5)		.001	
	Diabetes	756 (24.7)		349 (28.9)		.005	
	Hemiplegia or paraplegia	10 (0.3)		3 (0.2)		.91	
	Renal disease	449 (14.7)		210 (17.4)		.03	
	Malignancy	913 (29.8)		296 (24.5)		.001	
	HIV/AIDS	2 (0.1)		0 (0.0)		.92	
	Charlson Comorbidity Index	2 (1-4)		3 (2-4)		<.001	
**Laboratory tests**							
	Red blood cells (×10^9/L), median (IQR)	3.4 (2.9-3.9)		3.2 (2.7-3.8)		<.001	
	Hemoglobin (g/L), median (IQR)	103 (86-118)		96 (80-114)		<.001	
	RDW-CV^b^ (%), median (IQR)	14.0 (13.1-15.5)		15.0 (13.9-16.9)		<.001	
	White blood cells (×10^9/L), median (IQR)	9.8 (7.1-13.6)		11.4 (7.5-16.3)		<.001	
	Neutrophil percentage (%), median (IQR)	83.8 (75.6-89.3)		87.6 (80.8-91.9)		<.001	
	Lymphocyte percentage (%), median (IQR)	9.2 (5.6-15.1)		7.0 (4.1-11.9)		<.001	
	Platelets (×10^9/L), median (IQR)	175 (124-236)		147 (90-223)		<.001	
	Serum total protein (g/L), median (IQR)	58.4 (52.9-63.7)		56.5 (50.5-61.9)		<.001	
	Serum albumin (g/L), median (IQR)	32.3 (29.1-35.6)		30.0 (26.0-33.7)		<.001	
	Serum total bilirubin (μmol/L), median (IQR)	11.6 (7.9-18.4)		13.0 (8.0-23.4)		<.001	
	Serum direct bilirubin (μmol/L), median (IQR)	5.2 (3.3-8.6)		6.7 (3.9-14.1)		<.001	
	Alanine aminotransferase (U/L), median (IQR)	19.0 (11.5-34.6)		26.1 (13.4-68.2)		<.001	
	Aspartate aminotransferase (U/L), median (IQR)	26.5 (18.1-48.1)		42.3 (22.8-129.7)		<.001	
	Serum creatinine (μmol/L), median (IQR)	131.9 (97.7-186.7)		159.0 (109.4-240.2)		<.001	
	Blood urea nitrogen (mmol/L), median (IQR)	10.36 (7.16-15.36)		15.76 (10.79-22.96)		<.001	
	Blood uric acid (μmol/L), median (IQR)	331.3 (239.9-443.3)		385.2 (264.7-516.6)		<.001	
	Potassium (mmol/L), median (IQR)	4.2 (3.8-4.6)		4.3 (3.8-4.9)		<.001	
	Sodium (mmol/L), median (IQR)	139.5 (136.4-142.6)		141.0 (136.3-146.7)		<.001	
	Chloride (mmol/L), median (IQR)	103.2 (99.0-106.9)		103.1 (98.5-107.9)		.69	
	Calcium (mmol/L), median (IQR)	2.07 (1.96-2.20)		2.03 (1.90-2.17)		<.001	
	Mechanical ventilation, n (%)	442 (14.4)		564 (46.7)		<.001	
**Medications, n (%)**							
	Vasopressors	521 (17.0)		660 (54.7)		<.001	
	Diuretics	1699 (55.5)		849 (70.3)		<.001	
	ACEI^c^/ARB^d^	457 (14.9)		149 (12.3)		.03	
	NSAIDs^e^	1221 (39.9)		310 (25.7)		<.001	
	Proton pump inhibitors	2308 (75.4)		881 (73.0)		.10	
	Chemotherapeutic drugs	45 (1.5)		20 (1.7)		.76	
	Antiepileptic drugs	108 (3.5)		30 (2.5)		.10	
	Antituberculosis drugs	16 (0.5)		7 (0.6)		>.99	
	Nephrotoxic antibiotics	223 (7.3)		203 (16.8)		<.001	
	Antiviral drugs	41 (1.3)		35 (2.9)		.001	
	Antifungal drugs	186 (6.1)		232 (19.2)		<.001	
	Iodinated contrast media	159 (5.2)		50 (4.1)		.17	
**Outcomes**							
	Length of hospital stay (days), median (IQR)	8 (4-14)		7 (2-14)		<.001	
	Death within 30 days after discharge^f,g^, n (%)	201 (6.8)		137 (21.5)		<.001	
	Death within 1 year after discharge^f,g^, n (%)	637 (21.7)		247 (38.8)		<.001	

^a^MAKE30: Major adverse kidney events within 30 days.

^b^RDW-CV: red blood cell distribution width-coefficient of variation.

^c^ACEI: angiotensin converting enzyme inhibitor.

^d^ARB: angiotensin Ⅱ receptor blocker.

^e^NSAID: non-steroidal anti-inflammatory drug.

^f^There were 165 patients with missing postdischarge survival data.

^g^A total of 526 patients who died at hospital discharge or 30 days, whichever occurred first, were excluded.

### Model Performance

**Figure 2:**
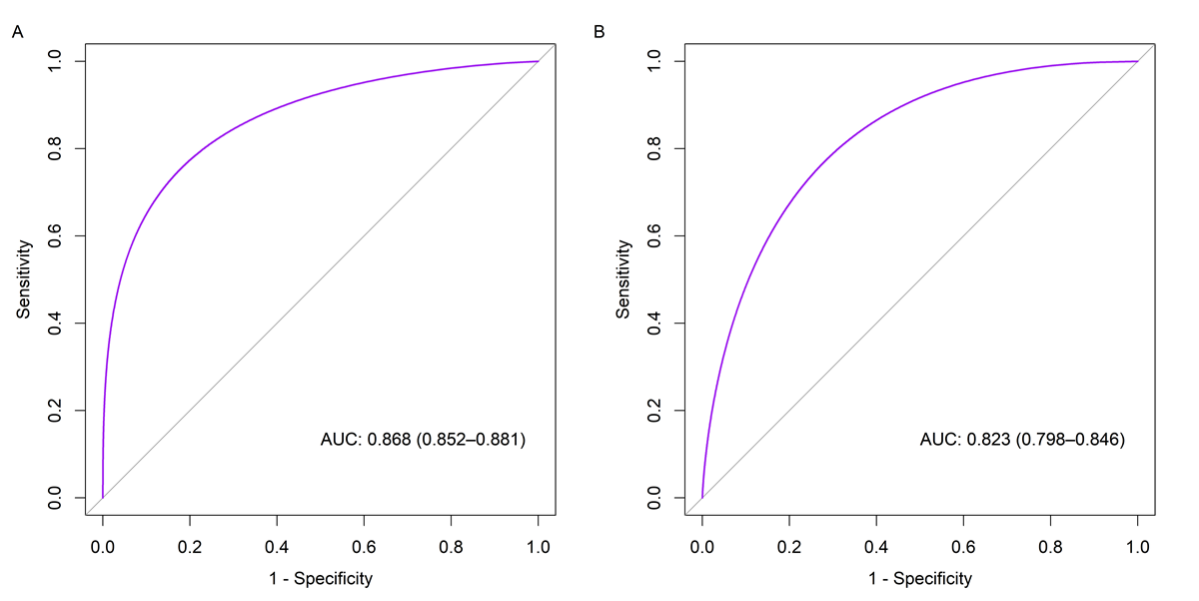
Receiver operating characteristic curves of the model in the training set (A) and internal test set (B). AUC: area under the curve.

**Figure 3:**
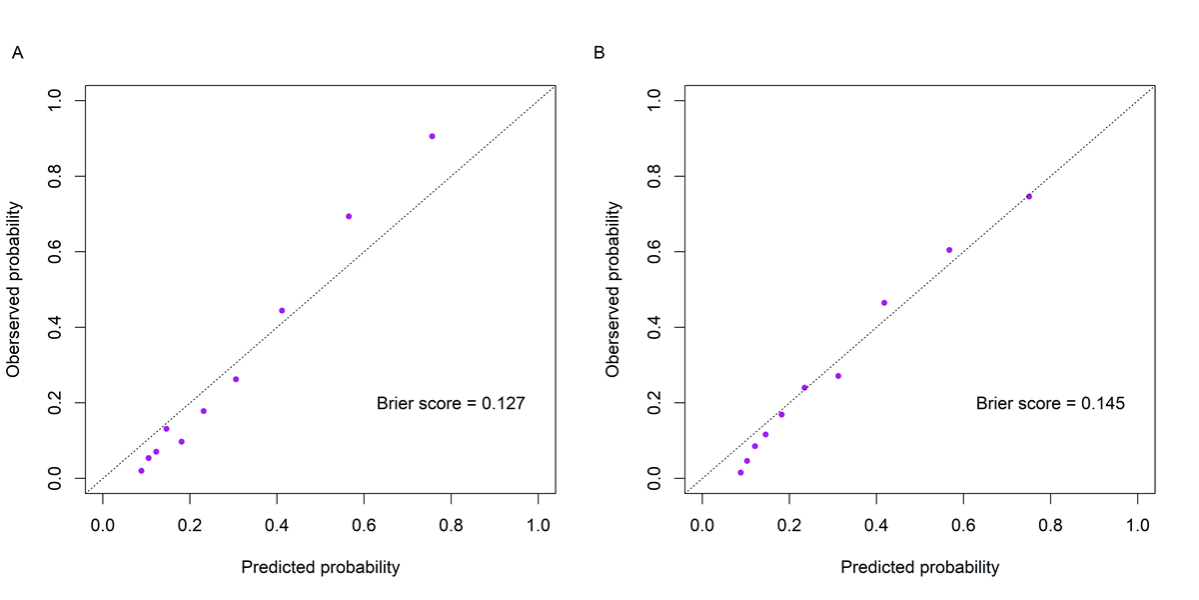
Calibration plots of the model in the training set (A) and internal test set (B).

**Figure 4:**
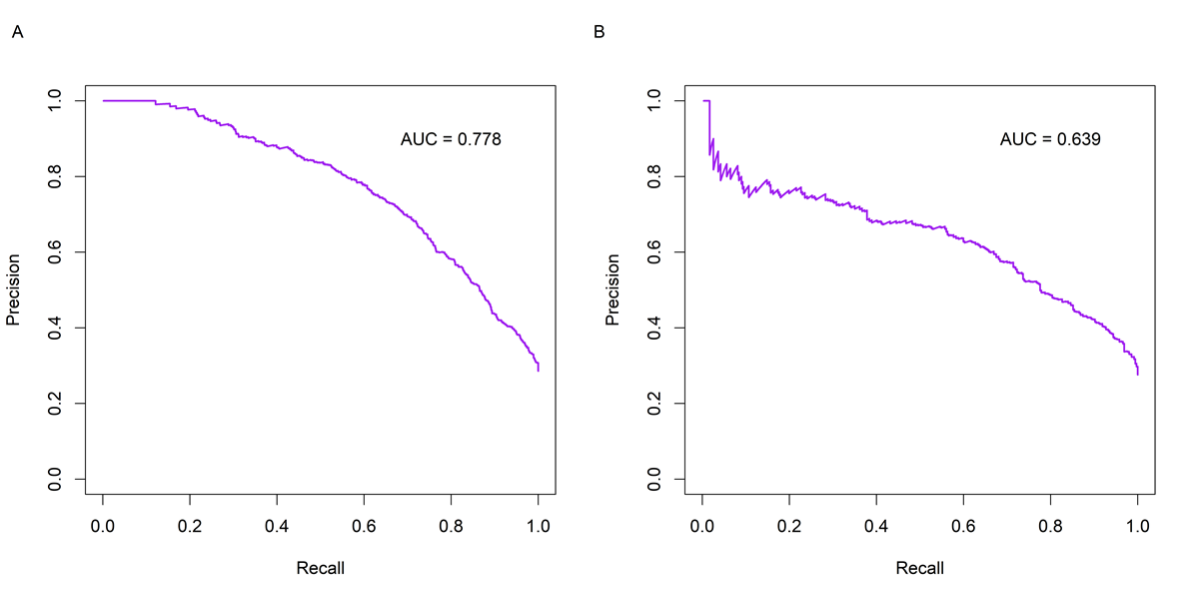
Precision-recall curves of the model in the training set (A) and internal test set (B). AUC: area under the curve.

**Figure 5:**
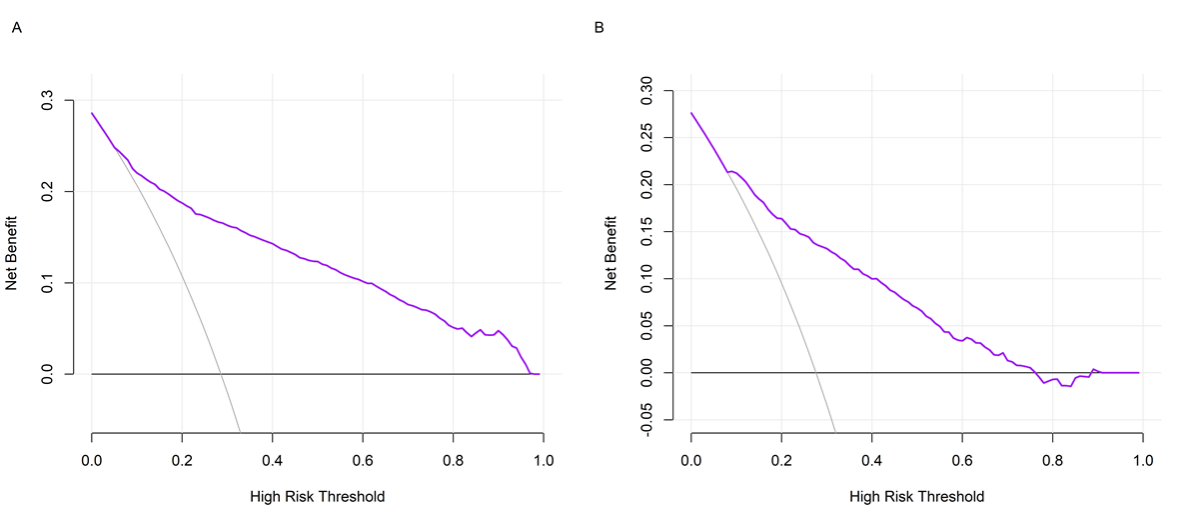
Decision curves of the model in the training set (A) and internal test set (B).

Figure 2 shows the ROC curves of the model in the training set and internal test set. The model exhibited good discrimination on both datasets, with the AUROC being 0.868 (95% CI 0.852-0.881) and 0.823 (95% CI 0.798-0.846), respectively. Figure 3 shows the calibration plots of the model in the training set and internal test set. The Brier scores on the 2 datasets were 0.127 and 0.145, respectively. Notably, despite the lower Brier score on the training set, the calibration plot exhibits pronounced deviations from the diagonal, especially at higher probability thresholds. In contrast, the calibration plot for the internal test set appears to demonstrate more consistent alignment with the ideal diagonal line across various probability ranges. Figure 4 shows the precision-recall curves of the model in the training set and internal test set. The model achieved an AUPRC of 0.778 and 0.639 on the 2 datasets, respectively. Figure 5 shows the decision curves of the model in the training set and internal test set. When the threshold probability was between 0.06 and 0.97, the model improved clinical decision-making in the training set. Similarly, within a narrower threshold probability range of 0.09 to 0.76, the model improved clinical decision-making in the internal test set. At the optimal cutoff of 0.350, the model achieved a sensitivity of 67.5%, a specificity of 82.5%, a PPV of 59.5%, and a NPV of 86.9% in the internal test set (Table 2).

Multimedia Appendix 8 presents the performance of the simplified model in the training set, the internal test set, and the external test set. It includes ROC curves, calibration plots, precision-recall curves, decision curves, and model performance at the optimal cutoff. The simplified model achieved an AUROC of 0.851 (95% CI 0.834-0.866) on the training set, 0.818 (95% CI 0.792-0.843) on the internal test set, and 0.744 (95% CI 0.735-0.754) on the external test set.

**Table 2:** Model performance at the optimal cutoff in the training and internal test sets.

Performance metrics	Training set	Internal test set
Cutoff value^a^	0.350	0.350
Sensitivity (%)	71.9	67.5
Specificity (%)	86.6	82.5
PPV^b^ (%)	68.3	59.5
NPV^c^ (%)	88.5	86.9
PLR^d^	5.37	3.86
NLR^e^	0.32	0.39

^a^The optimal cutoff was determined by the receiver operating characteristic curve and the maximum Youden index (sensitivity + specificity – 1) in the training set.

^b^PPV: positive predictive value.

^c^NPV: negative predictive value.

^d^PLR, positive likelihood ratio.

^e^NLR, negative likelihood ratio.

### Model Interpretations

**Figure 6:**
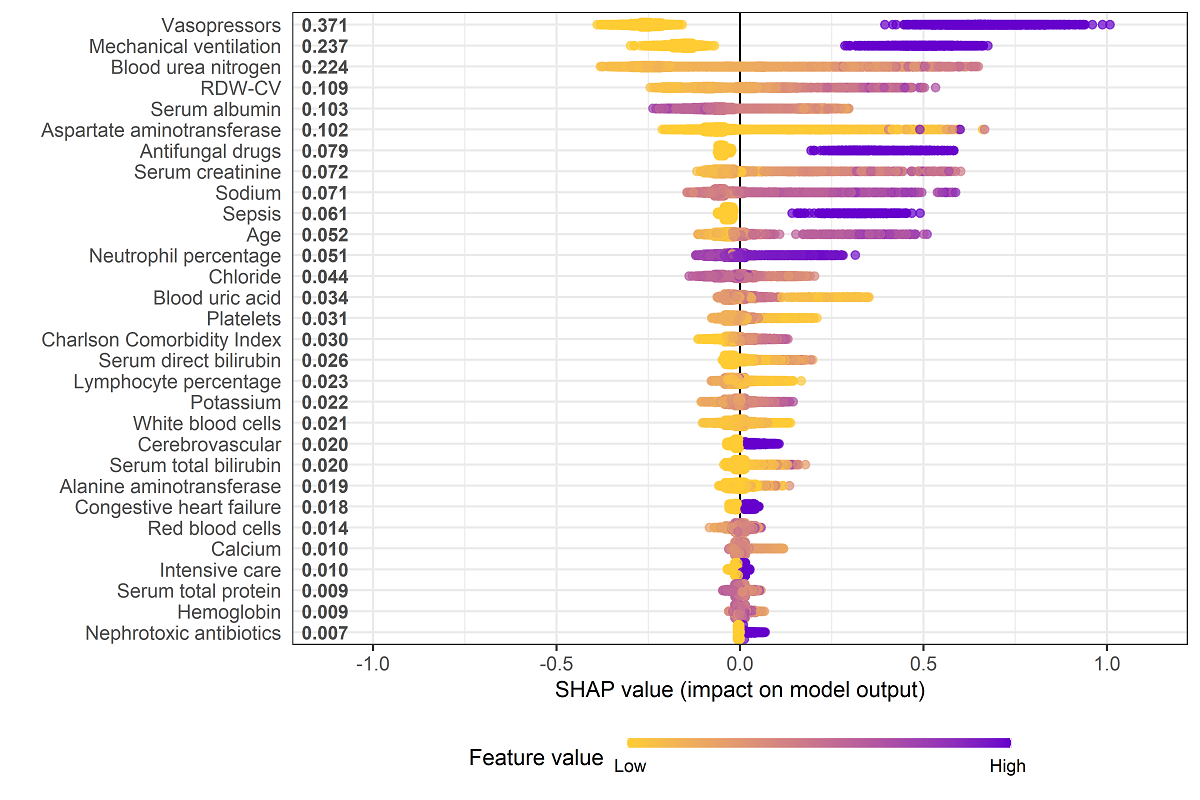
SHapley Additive exPlanations summary plot of the model. RDW-CV: red blood cell distribution width-coefficient of variation.

Figure 6 shows the SHAP summary plots of the model. The use of vasopressors, requirement for mechanical ventilation, increased blood urea nitrogen level, increased red blood cell distribution width coefficient of variation, decreased serum albumin level, elevated aspartate aminotransferase level, use of antifungal drugs, and increased SCr level were identified as important predictor variables associated with an increased risk of MAKE30. Multimedia Appendix 9 provides the SHAP dependence plots of the model that visualize how changes in each variable can affect model output.

### Sensitivity Analyses

Multimedia Appendix 10 shows the ROC curves of the model and the simplified model in distinct age groups. In the internal test set, the model achieved AUROCs of 0.827 (95% CI 0.786-0.862), 0.835 (95% CI 0.794-0.869), and 0.770 (95% CI 0.708-0.837) in patients aged <70 years, 70 to 80 years, and ≥80 years, respectively. When assessing the simplified model across distinct age groups in the external test set, its performance exhibited overall stability.

## Discussion

### Principal Findings

This study showed that MAKE30 is common in hospitalized older patients with AKI. The main deliverable of this study is the development and validation of a machine learning–based model to predict MAKE30, representing a pioneering effort to address the critical need in this vulnerable population. The XGBoost model (or its simplified version) achieved adequate predictive performance in both internal and external validation. The model could be a useful tool for prognostic assessment, clinical decisionmaking, and the conduct of clinical trials involving AKI.

A National Institute of Diabetes and Digestive and Kidney Diseases Workshop recommended a composite of death, provision of dialysis, or sustained loss of kidney function at a discrete time point as a meaningful endpoint for trials [9]. This endpoint was later expanded to be MAKE30, which occurred at hospital discharge truncated at 30 days [34,35]. MAKE30 has been shown to be common, easily measurable, and a promising endpoint for patients with AKI. Our study further confirmed that MAKE30 is a prevalent endpoint among hospitalized older patients with AKI, with its nonfatal components being associated with long-term outcomes.

The prediction of MAKE30 is essential for risk stratification and clinical management of hospitalized older patients with AKI. While several prediction models for MAKE30 have been developed, none specifically focus on this population. A recent study developed a logistic regression model specifically for predicting MAKE30 among critically ill adults [36]. Our previous studies established machine learning–based models to predict MAKE30 in hospitalized children with AKI [37] and older patients in critical care [38]. To the best of our knowledge, this study is the first to use machine learning algorithm to predict MAKE30 in hospitalized older patients with AKI. This demographic often experiences impaired recovery of kidney function, resulting in significantly increased morbidity and mortality. By developing a prediction model tailored to this population, we aimed to enhance clinical outcomes and facilitate more effective interventions. The model can be seamlessly integrated into electronic medical record systems, assisting health care providers in risk stratification and informed clinical decision-making. In addition, in clinical trials involving AKI, the model can identify high-risk groups that are more likely to benefit from the intervention.

Compared with the existing studies, this study has several strengths. First, by using state-of-the-art XGBoost machine learning algorithm, our model represented a significant advancement over previous studies that primarily relied on clinical rationale and simpler statistical methods. XGBoost is particularly effective due to its ability to process large amounts of data and identify intricate patterns within complex datasets. Its gradient boosting framework enhances predictive accuracy by combining the strengths of multiple weak learners, leading to more robust and reliable outcomes. Furthermore, XGBoost’s flexibility allows for easy tuning of hyperparameters, enabling us to optimize the model for our specific dataset and improve its performance in predicting MAKE30 in hospitalized older patients with AKI.

Second, this study used a larger sample size and conducted external validation to evaluate the model’s performance. Notably, the results of the external validation showed that the simplified version of the model exhibited a gap of approximately 10% in AUROC between the training set and the external test set. However, the performance of the simplified model on the internal test set was comparable to that of the full model, suggesting that the observed gap may be attributed to the use of a different test set with distinct characteristics. Compared with the training set, the external test set comprised critically ill patients from various ethnic backgrounds and with distinct patient characteristics and data quality. These findings underscore the necessity for comprehensive testing across diverse datasets to ensure the model’s reliability and applicability in real-world settings. Another noteworthy observation is that, although the Brier scores for both the training and test data suggested acceptable calibration, a deeper examination through visual inspection uncovered disparities. Factors contributing to the observed discrepancies may include overfitting during training, differences in underlying data distributions, model complexity, or nuances in evaluation methodology. Further investigation into the factors contributing to the observed discrepancies is warranted.

Finally, this study delved into the interpretability of the model. A major challenge in the clinical implementation of machine learning is how to uncover its “black box” nature. Usually, data come in and decisions go out, but the process between input and output is opaque. The advantage of our study is the use of SHAP method to explain the critical aspects of the data and fully understand the model. The SHAP method identified important predictor variables associated with the development of MAKE30, which could allow early intervention of modifiable factors to mitigate the risk of MAKE30. Another notable finding is the nonlinear relationship between the predictor variables and the risk of MEKE30, as shown in the SHAP dependence plots. This relationship is often ignored by traditional regression analysis, which requires a linearity between the independent variables and the outcome.

### Limitations

Our study has several limitations. First, the MIMIC-Ⅳ database only contained data from critically ill patients admitted to the ICU. Model performance awaits further validation in larger samples of patients at different medical centers. Second, it remains unclear whether the model will perform well in individual prognostication and whether its clinical application will improve patient prognosis. Thus, clinical impact studies are needed to confirm the effectiveness of the model. Third, the unavailability of preadmission SCr data could lead to the omission of patients who could have been diagnosed with AKI, that is, community-acquired AKI. Fourth, the urine output criteria were not used to define AKI because hourly urine output data were unavailable for most patients. Finally, the missForest method, as a single imputation method, might affect the SHAP results by not considering sufficient variation for the imputation of missing data.

### Conclusions

MAKE30 is common in hospitalized older patients with AKI. An interpretable machine learning XGBoost model was developed and validated to predict MAKE30. The model exhibited adequate predictive performance, which provides opportunities for risk stratification, clinical decision-making, and the conduct of clinical trials involving AKI. Future studies are needed to support the robustness and clinical effectiveness of the model.

## Appendix

Multimedia Appendix 1 The list of 53 candidate predictor variables and 30 variables identified in feature selection.

Multimedia Appendix 2 Missing data in predictor variables.

Multimedia Appendix 3 The descriptions and search ranges for the hyperparameters of the XGBoost (eXtreme Gradient Boosting) model.

Multimedia Appendix 4 Flow diagram of patient selection from the MIMIC-Ⅳ (Medical Information Mart for Intensive Care Ⅳ) database.

Multimedia Appendix 5 Kaplan-Meier survival curves of death within 30 days and 1 year for patients alive at discharge from the Second Xiangya Hospital.

Multimedia Appendix 6 Characteristics of patients from the MIMIC-Ⅳ (Medical Information Mart for Intensive Care Ⅳ) database.

Multimedia Appendix 7 Characteristics of patients in the training set, internal test set, and external test set.

Multimedia Appendix 8 Performance of the simplified model in the training set, internal test set, and external test set.

Multimedia Appendix 9 SHAP (SHapley Additive exPlanations) dependence plots of the model.

Multimedia Appendix 10 Receiver operating characteristic curves of the model and the simplified model in distinct age groups.

## Abbreviations

AKI: Acute kidney injury
AUPRC: area under the precision-recall curve
AUROC: area under the receiver operating characteristic curve
BIDMC: Beth Israel Deaconess Medical Center
EHR: electronic health record
ICD-9: International Classification of Diseases, Ninth Revision
ICD-10: International Statistical Classification of Diseases, Tenth Revision
ICU: intensive care unit
MAKE30: Major adverse kidney events within 30 days
MIMIC-Ⅳ: Medical Information Mart for Intensive Care Ⅳ
NLR: negative likelihood ratio
NPV: negative predictive value
PLR: positive likelihood ratio
PPV: positive predictive value
PRD: persistent renal dysfunction
ROC: receiver operating characteristic
RRT: renal replacement therapy
SCr: serum creatinine
SHAP: SHapley Additive exPlanations
XGBoost: eXtreme Gradient Boosting
