# Supplementary material for: Major Adverse Kidney Events in Hospitalized Older Patients With Acute Kidney Injury: Machine Learning–Based Model Development and Validation Study
Source: J Med Internet Res. 2025 Jan 3;27:e52786. doi: 10.2196/52786 (PMC11748444; doi:10.2196/52786)
Supplement: Multimedia Appendix 1 [file jmir_v27i1e52786_app1.docx]

The list of 53 candidate predictor variables:

Age, Sex, Intensive care, Sepsis, Hypertension, Myocardial infarction, Congestive heart failure, Peripheral vascular disease, Cerebrovascular disease, Dementia, Chronic pulmonary disease, Rheumatic disease, Peptic ulcer disease, Liver disease, Diabetes, Hemiplegia or paraplegia, Renal disease, Malignancy, HIV/AIDS, Charlson Comorbidity Index, Red blood cells, Hemoglobin, RDW-CV, White blood cells, Neutrophil percentage, Lymphocyte percentage, Platelets, Serum total protein, Serum albumin, Serum total bilirubin, Serum direct bilirubin, Alanine aminotransferase, Aspartate aminotransferase, Serum creatinine, Blood urea nitrogen, Blood uric acid, Potassium, Sodium, Chloride, Calcium, Mechanical ventilation, Vasopressors, Diuretics, ACEI/ARB, NSAIDs, Proton pump inhibitors, Chemotherapeutic drugs, Antiepileptic drugs, Antituberculosis drugs, Nephrotoxic antibiotics, Antiviral drugs, Antifungal drugs, Iodinated contrast media

The list of 30 variables identified in feature selection:

Age, Intensive care, Sepsis, Congestive heart failure, Cerebrovascular disease, Charlson Comorbidity Index, Red blood cells, Hemoglobin, RDW-CV, White blood cells, Neutrophil percentage, Lymphocyte percentage, Platelets, Serum total protein, Serum albumin, Serum total bilirubin, Serum direct bilirubin, Alanine aminotransferase, Aspartate aminotransferase, Serum creatinine, Blood urea nitrogen, Blood uric acid, Potassium, Sodium, Chloride, Calcium, Mechanical ventilation, Vasopressors, Nephrotoxic antibiotics, Antifungal drugs
