# Supplementary material for: Major Adverse Kidney Events in Hospitalized Older Patients With Acute Kidney Injury: Machine Learning–Based Model Development and Validation Study
Source: J Med Internet Res. 2025 Jan 3;27:e52786. doi: 10.2196/52786 (PMC11748444; doi:10.2196/52786)
Supplement: Multimedia Appendix 2 [file jmir_v27i1e52786_app2.docx]

Missing data in predictor variables.

| Variables | Training set | Internal test set | External test set |
| --- | --- | --- | --- |
| Age | 0 (0.0) | 0 (0.0) | 0 (0.0) |
| Intensive care | 0 (0.0) | 0 (0.0) | 0 (0.0) |
| Sepsis | 0 (0.0) | 0 (0.0) | 0 (0.0) |
| Congestive heart failure | 0 (0.0) | 0 (0.0) | 0 (0.0) |
| Cerebrovascular disease | 0 (0.0) | 0 (0.0) | 0 (0.0) |
| Charlson Comorbidity Index | 0 (0.0) | 0 (0.0) | 0 (0.0) |
| Red blood cells | 220 (7.4) | 105 (8.1) | 131 (1.1) |
| Hemoglobin | 220 (7.4) | 105 (8.1) | 128 (1.1) |
| RDW-CV | 222 (7.5) | 108 (8.4) | 133 (1.1) |
| White blood cells | 220 (7.4) | 105 (8.1) | 128 (1.1) |
| Neutrophil percentage | 228 (7.7) | 114 (8.8) | 8538 (72.0) |
| Lymphocyte percentage | 227 (7.6) | 112 (8.7) | 8537 (72.0) |
| Platelets | 221 (7.4) | 105 (8.1) | 134 (1.1) |
| Serum total protein | 447 (15.0) | 185 (14.3) | 11660 (98.3) |
| Serum albumin | 387 (13.0) | 161 (12.5) | 8673 (73.1) |
| Serum total bilirubin | 457 (15.4) | 185 (14.3) | 7037 (59.3) |
| Serum direct bilirubin | 456 (15.3) | 186 (14.4) | 11350 (95.7) |
| Alanine aminotransferase | 437 (14.7) | 181 (14.0) | 7110 (59.9) |
| Aspartate aminotransferase | 442 (14.9) | 184 (14.2) | 7063 (59.5) |
| Serum creatinine | 0 (0.0) | 0 (0.0) | 0 (0.0) |
| Blood urea nitrogen | 14 (0.5) | 6 (0.5) | 2 (0.02) |
| Blood uric acid | 35 (1.2) | 10 (0.8) | 11538 (97.3) |
| Potassium | 124 (4.2) | 62 (4.8) | 3 (0.03) |
| Sodium | 128 (4.3) | 62 (4.8) | 8 (0.07) |
| Chloride | 130 (4.4) | 64 (4.9) | 17 (0.1) |
| Calcium | 132 (4.4) | 62 (4.8) | 968 (8.2) |
| Mechanical ventilation | 0 (0.0) | 0 (0.0) | 0 (0.0) |
| Vasopressors | 0 (0.0) | 0 (0.0) | 0 (0.0) |
| Nephrotoxic antibiotics | 0 (0.0) | 0 (0.0) | 0 (0.0) |
| Antifungal drugs | 0 (0.0) | 0 (0.0) | 0 (0.0) |

RDW-CV, red blood cell distribution width-coefficient of variation.
