# Supplementary material for: Major Adverse Kidney Events in Hospitalized Older Patients With Acute Kidney Injury: Machine Learning–Based Model Development and Validation Study
Source: J Med Internet Res. 2025 Jan 3;27:e52786. doi: 10.2196/52786 (PMC11748444; doi:10.2196/52786)
Supplement: Multimedia Appendix 3 [file jmir_v27i1e52786_app3.docx]

The descriptions and search ranges for the hyperparameters of the XGBoost model.

| Hyperparameters | Descriptions | Search ranges |
| --- | --- | --- |
| eta | Learning rate | 0.01, 0.05, 0.1, 0.2, 0.3 |
| max_depth | Maximum depth of a tree | 3, 4, 5, 6, 7, 8, 9, 10 |
| min_child_weight | Minimum sum of instance weight (hessian) needed in a child | 1, 2, 3, 4, 5, 6 |
| gamma | Minimum loss reduction required to make a further partition on a leaf node of the tree | 0, 0.1, 0.2, 0.3, 0.4, 0.5 |
| colsample_bytree | Subsample ratio of columns when constructing each tree | 0.5, 0.6, 0.7, 0.8, 0.9 |
| subsample | Subsample ratio of the training instances | 0.5, 0.6, 0.7, 0.8, 0.9 |

The final values for each hyperparameters of the model are as follows: eta = 0.01, max_depth = 4, min_child_weight = 5, gamma = 0.3, colsample_bytree = 0.8, and subsample = 0.5.

The final values for each hyperparameters of the simplified model are as follows: eta = 0.01, max_depth = 4, min_child_weight = 3, gamma = 0.2, colsample_bytree = 0.5, and subsample = 0.5.
