# Supplementary material for: Major Adverse Kidney Events in Hospitalized Older Patients With Acute Kidney Injury: Machine Learning–Based Model Development and Validation Study
Source: J Med Internet Res. 2025 Jan 3;27:e52786. doi: 10.2196/52786 (PMC11748444; doi:10.2196/52786)

Flow diagram of patient selection from the MIMIC-Ⅳ database. AKI, acute kidney injury; MIMIC-Ⅳ, Medical Information Mart for Intensive Care Ⅳ; SCr, serum creatinine; RRT, renal replacement therapy; MAKE30, major adverse kidney events within 30 days.


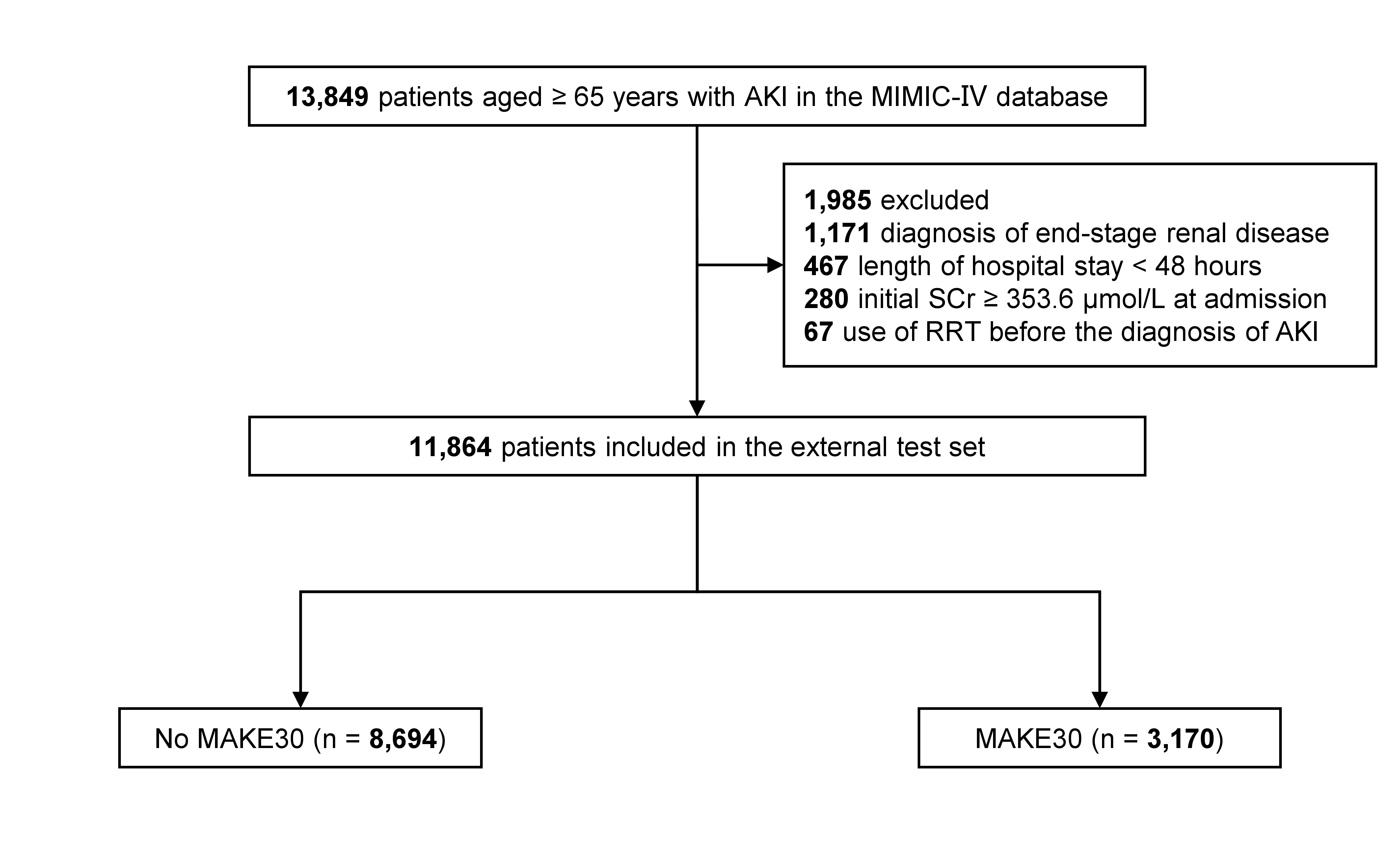

Supplement: Multimedia Appendix 4 [file jmir_v27i1e52786_app4.docx]
