# Supplementary material for: Major Adverse Kidney Events in Hospitalized Older Patients With Acute Kidney Injury: Machine Learning–Based Model Development and Validation Study
Source: J Med Internet Res. 2025 Jan 3;27:e52786. doi: 10.2196/52786 (PMC11748444; doi:10.2196/52786)

Kaplan-Meier survival curves of death within 30 days (A) and 1 year (B) for patients alive at discharge from the Second Xiangya Hospital stratified by PRD. There were 165 patients with missing post-discharge survival data. PRD, persistent renal dysfunction.


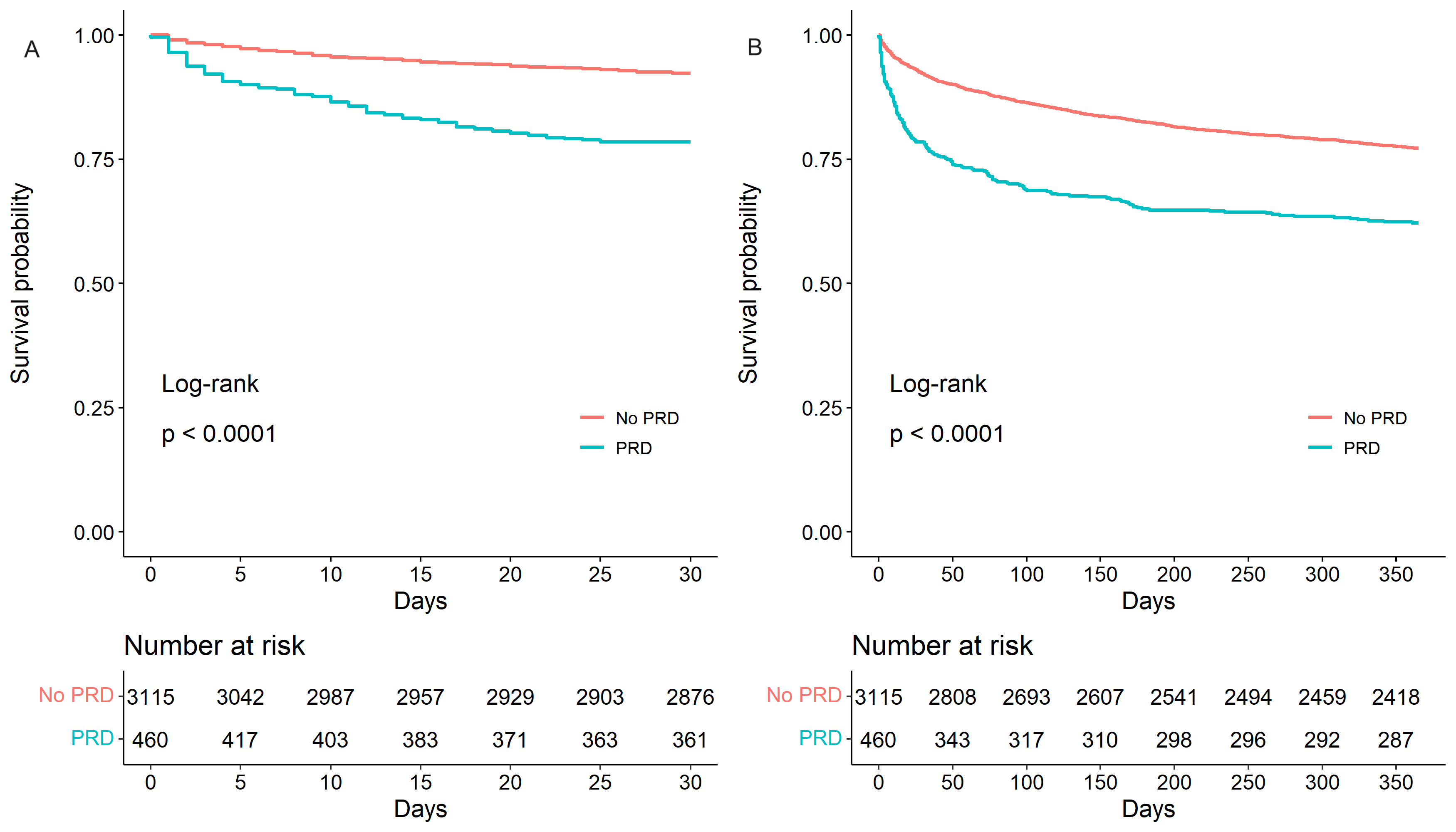


Kaplan-Meier survival curves of death within 30 days (A) and 1 year (B) for patients alive at discharge from the Second Xiangya Hospital stratified by RRT. There were 165 patients with missing post-discharge survival data. RRT, renal replacement therapy.


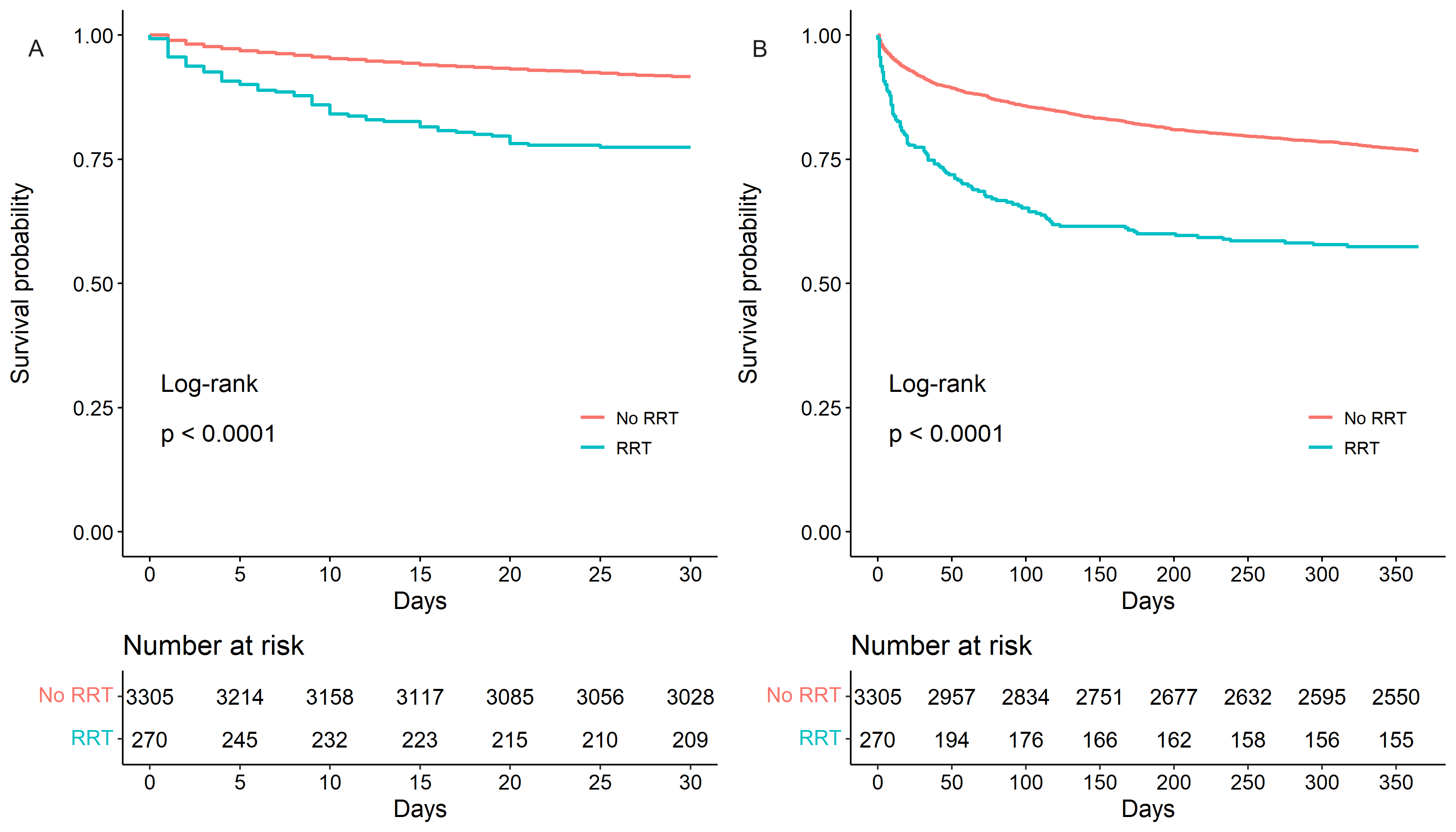

Supplement: Multimedia Appendix 5 [file jmir_v27i1e52786_app5.docx]
