# Supplementary material for: Major Adverse Kidney Events in Hospitalized Older Patients With Acute Kidney Injury: Machine Learning–Based Model Development and Validation Study
Source: J Med Internet Res. 2025 Jan 3;27:e52786. doi: 10.2196/52786 (PMC11748444; doi:10.2196/52786)
Supplement: Multimedia Appendix 6 [file jmir_v27i1e52786_app6.docx]

Characteristics of patients from the MIMIC-Ⅳ database.

| Variables | No MAKE30  (n = 8,694) | MAKE30  (n = 3,170) | *P* value |
| --- | --- | --- | --- |
| Age (year) | 77 (71-83) | 78 (71-85) | <0.001 |
| Intensive care, n (%) | 8694 (100.0) | 3170 (100.0) | - |
| Sepsis, n (%) | 1078 (12.4) | 1154 (36.4) | <0.001 |
| Congestive heart failure, n (%) | 3745 (43.1) | 1554 (49.0) | <0.001 |
| Cerebrovascular disease, n (%) | 1406 (16.2) | 587 (18.5) | 0.003 |
| Charlson Comorbidity Index | 3 (1-5) | 4 (2-6) | <0.001 |
| Red blood cells (× 10^9/L) | 3.4 (3.0-3.8) | 3.3 (2.8-3.8) | <0.001 |
| Hemoglobin (g/L) | 100 (88-113) | 96 (84-111) | <0.001 |
| RDW-CV (%) | 14.8 (13.8-16.2) | 15.7 (14.4-17.5) | <0.001 |
| White blood cells (× 10^9/L) | 10.8 (8.0-14.5) | 11.6 (8.1-16.2) | <0.001 |
| Platelets (× 10^9/L) | 184 (130-259) | 187 (121-266) | 0.20 |
| Serum creatinine (μmol/L) | 114.9 (88.4-150.3) | 132.6 (88.4-176.8) | <0.001 |
| Blood urea nitrogen (mmol/L) | 8.93 (6.43-12.86) | 11.07 (7.14-16.79) | <0.001 |
| Potassium (mmol/L) | 4.3 (3.9-4.7) | 4.3 (3.8-4.7) | 0.70 |
| Sodium (mmol/L) | 138 (135-141) | 139 (135-142) | <0.001 |
| Chloride (mmol/L) | 102 (98-106) | 103 (98-108) | <0.001 |
| Calcium (mmol/L) | 2.12 (2.02-2.22) | 2.08 (1.95-2.22) | <0.001 |
| Mechanical ventilation, n (%) | 1890 (21.7) | 1516 (47.8) | <0.001 |
| Vasopressors, n (%) | 1721 (19.8) | 1348 (42.5) | <0.001 |
| Nephrotoxic antibiotics, n (%) | 3552 (40.9) | 1846 (58.2) | <0.001 |
| Antifungal drugs, n (%) | 644 (7.4) | 395 (12.5) | <0.001 |

Continuous variables were presented as median (interquartile range) and categorical variables were presented as n (%).

MAKE30, major adverse kidney events within 30 days; RDW-CV, red blood cell distribution width-coefficient of variation.
