# Supplementary material for: Major Adverse Kidney Events in Hospitalized Older Patients With Acute Kidney Injury: Machine Learning–Based Model Development and Validation Study
Source: J Med Internet Res. 2025 Jan 3;27:e52786. doi: 10.2196/52786 (PMC11748444; doi:10.2196/52786)
Supplement: Multimedia Appendix 7 [file jmir_v27i1e52786_app7.docx]

Characteristics of patients in the training, internal test, and external test sets.

| Variables | Training set  (n = 2,973) | Internal test set  (n = 1,293) | External test set  (n = 14,083) |
| --- | --- | --- | --- |
| Age (year) | 72 (68-78) | 72 (68-78) | 77 (71-84) |
| Intensive care, n (%) | 1416 (47.6) | 633 (49.0) | 11864 (100.0) |
| Sepsis, n (%) | 252 (8.5) | 112 (8.7) | 2232 (18.8) |
| Congestive heart failure, n (%) | 993 (33.4) | 441 (34.1) | 5299 (44.7) |
| Cerebrovascular disease, n (%) | 669 (22.5) | 289 (22.4) | 1993 (16.8) |
| Charlson Comorbidity Index | 2 (1-4) | 2 (1-4) | 3 (2-5) |
| Red blood cells (× 10^9/L) | 3.4 (2.9-3.9) | 3.4 (2.8-3.9) | 3.3 (2.9-3.8) |
| Hemoglobin (g/L) | 101 (84-117) | 101 (84-117) | 99 (87-113) |
| RDW-CV (%) | 14.3 (13.2-15.9) | 14.2 (13.2-15.8) | 15.0 (13.9-16.5) |
| White blood cells (× 10^9/L) | 10.2 (7.2-14.2) | 10.0 (7.2-14.6) | 11.0 (8.0-14.8) |
| Neutrophil percentage (%) | 84.8 (76.8-90.1) | 85.0 (76.7-90.0) | - |
| Lymphocyte percentage (%) | 8.4 (5.0-14.4) | 8.8 (5.2-14.3) | - |
| Platelets (× 10^9/L) | 168 (110-231) | 174 (121-239) | 185 (128-261) |
| Serum total protein (g/L) | 57.7 (52.2-62.9) | 58.0 (52.4-64.2) | - |
| Serum albumin (g/L) | 31.7 (28.1-35.2) | 31.8 (28.2-35.3) | - |
| Serum total bilirubin (μmol/L) | 12.1 (8.0-19.5) | 11.8 (7.6-20.1) | - |
| Serum direct bilirubin (μmol/L) | 5.5 (3.5-9.7) | 5.3 (3.4-9.7) | - |
| Alanine aminotransferase (U/L) | 20.4 (11.9-42.3) | 21.0 (12.4-40.7) | - |
| Aspartate aminotransferase (U/L) | 28.9 (19.0-59.1) | 29.9 (19.5-61.3) | - |
| Serum creatinine (μmol/L) | 139.0 (99.1-197.8) | 139.3 (101.3-198.6) | 114.9 (88.4-159.1) |
| Blood urea nitrogen (mmol/L) | 11.78 (7.86-17.61) | 11.37 (7.70-17.44) | 9.29 (6.79-13.93) |
| Blood uric acid (μmol/L) | 344.3 (244.4-461.8) | 347.9 (248.1-465.4) | - |
| Potassium (mmol/L) | 4.2 (3.8-4.6) | 4.2 (3.8-4.7) | 4.3 (3.9-4.7) |
| Sodium (mmol/L) | 139.6 (136.3-143.5) | 140.0 (136.5-143.8) | 138.0 (135.0-141.0) |
| Chloride (mmol/L) | 103.1 (98.6-107.0) | 103.5 (99.2-107.5) | 102.0 (98.0-106.0) |
| Calcium (mmol/L) | 2.07 (1.94-2.19) | 2.07 (1.93-2.18) | 2.12 (2.00-2.22) |
| Mechanical ventilation, n (%) | 684 (23.0) | 322 (24.9) | 3406 (28.7) |
| Vasopressors, n (%) | 811 (27.3) | 370 (28.6) | 3069 (25.9) |
| Nephrotoxic antibiotics, n (%) | 292 (9.8) | 134 (10.4) | 5398 (45.5) |
| Antifungal drugs, n (%) | 295 (9.9) | 123 (9.5) | 1039 (8.8) |

Continuous variables were presented as median (interquartile range) and categorical variables were presented as n (%).

RDW-CV, red blood cell distribution width-coefficient of variation.
