# Supplementary material for: Major Adverse Kidney Events in Hospitalized Older Patients With Acute Kidney Injury: Machine Learning–Based Model Development and Validation Study
Source: J Med Internet Res. 2025 Jan 3;27:e52786. doi: 10.2196/52786 (PMC11748444; doi:10.2196/52786)
Supplement: Multimedia Appendix 8 [file jmir_v27i1e52786_app8.docx]

Receiver operating characteristic curves of the simplified model in the training set (A), internal test set (B), and external test set (C). AUC, area under the curve.


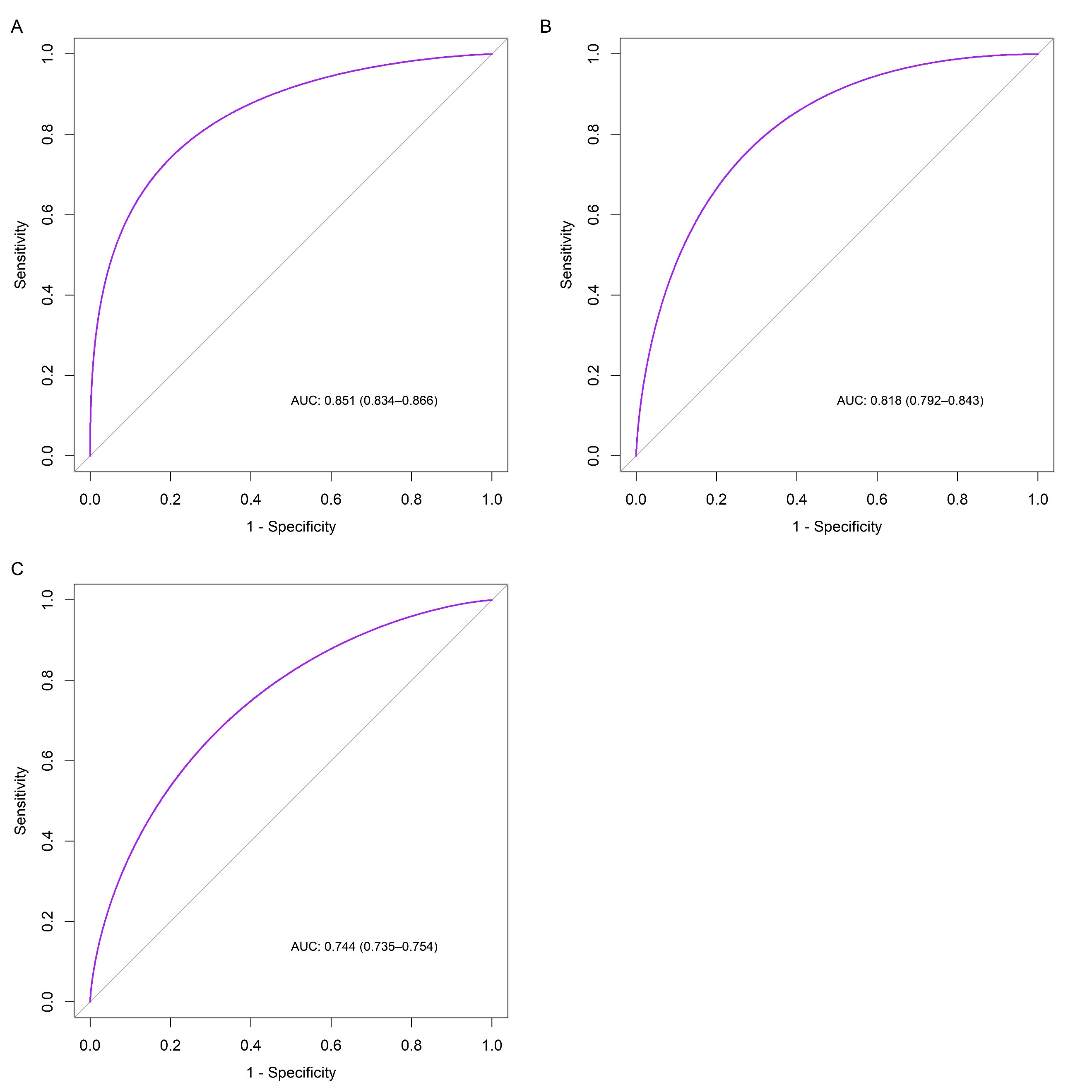


Calibration curves of the simplified model in the training set (A), internal test set (B), and external test set (C).


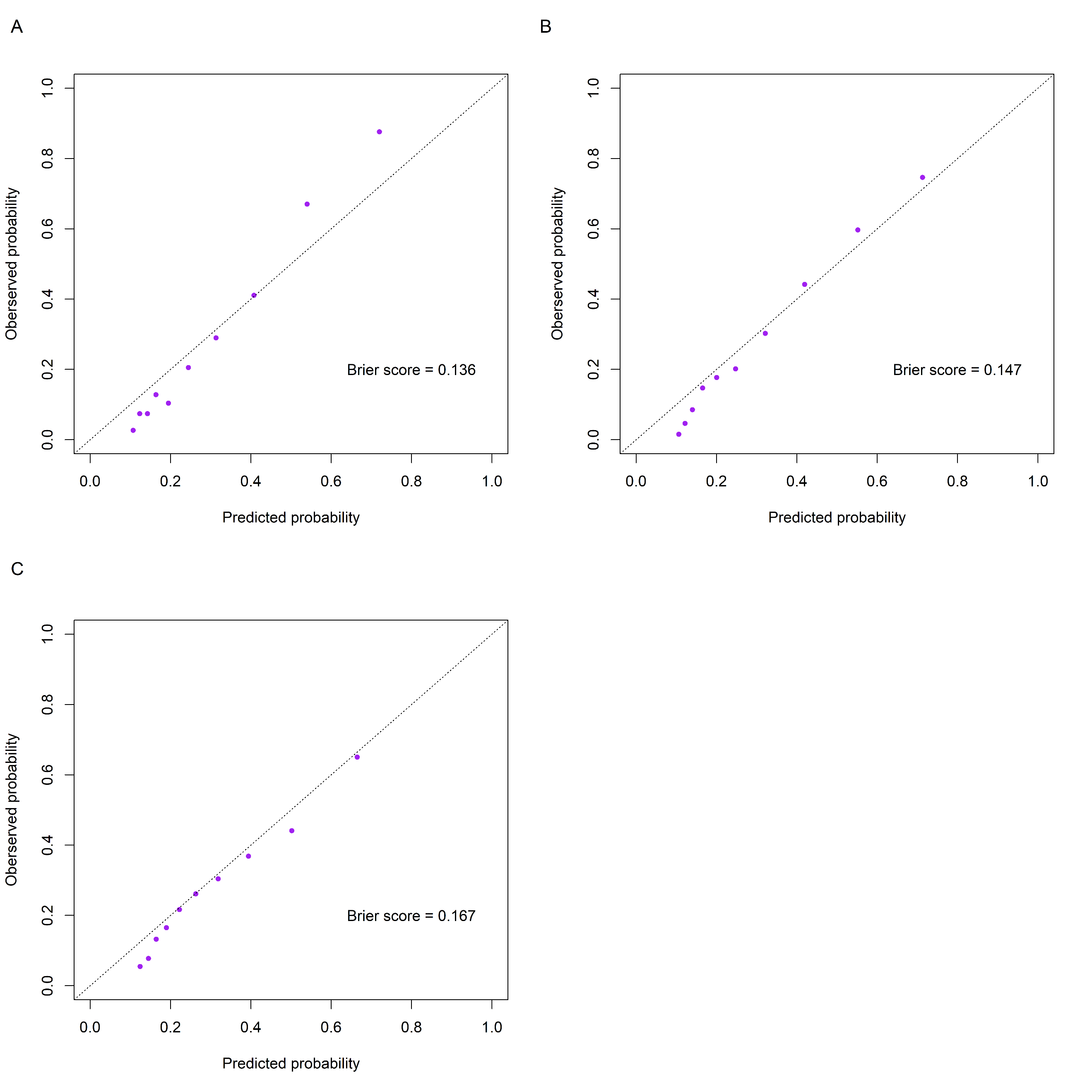


Precision-recall curves of the simplified model in the training set (A), internal test set (B), and external test set (C). AUC, area under the curve.


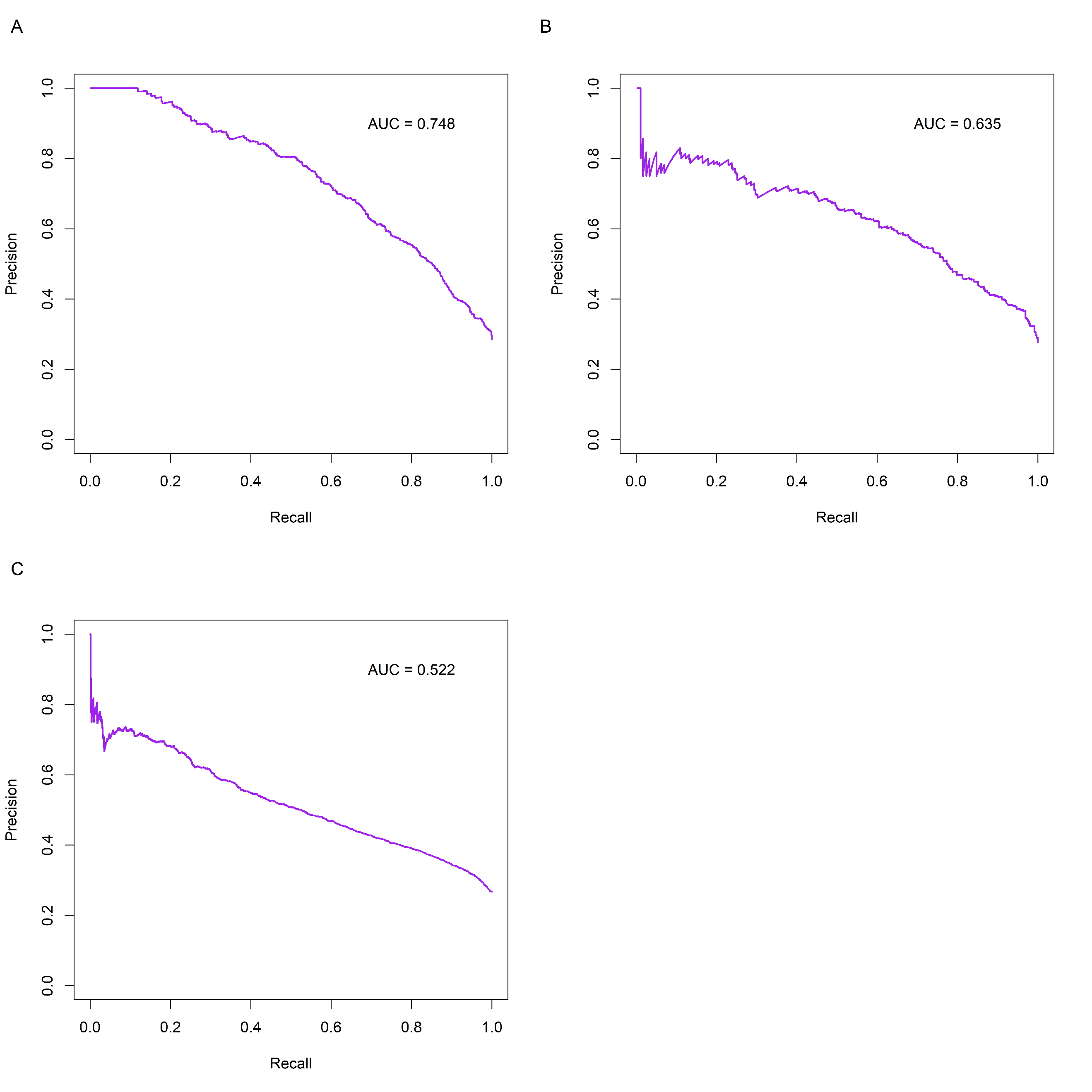


Decision curves of the simplified model in the training set (A), internal test set (B), and external test set (C).


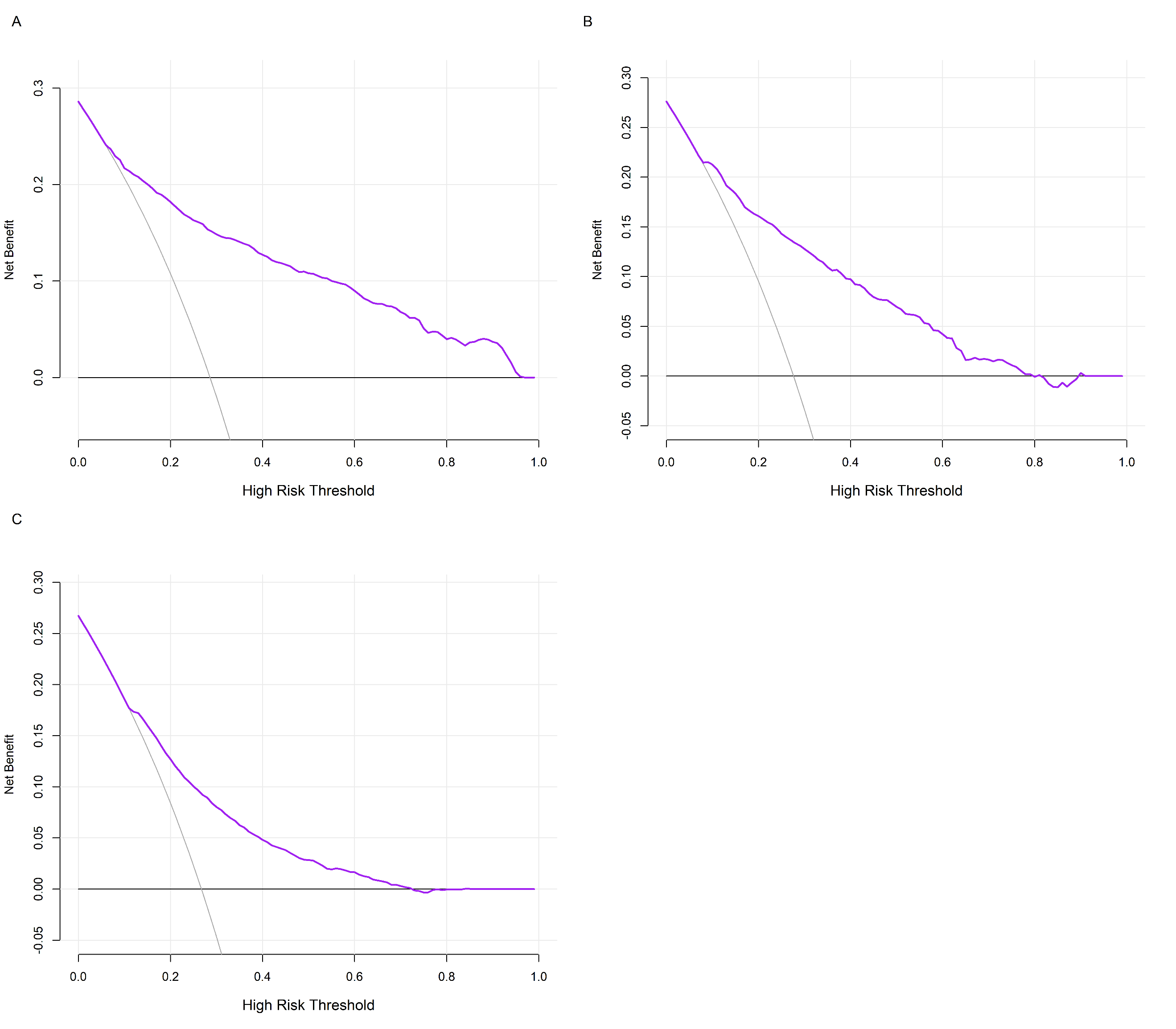


Model performance at the optimal cutoff in the training set, internal test set, and external test set.

| Performance metrics | Training set | Internal test set | External test set |
| --- | --- | --- | --- |
| Cutoff value^*^ | 0.317 | 0.317 | 0.317 |
| Sensitivity (%) | 73.3 | 70.9 | 61.0 |
| Specificity (%) | 81.1 | 78.1 | 74.3 |
| PPV (%) | 60.8 | 55.2 | 46.4 |
| NPV (%) | 88.3 | 87.5 | 83.9 |
| PLR | 3.88 | 3.24 | 2.37 |
| NLR | 0.33 | 0.37 | 0.52 |

PPV, positive predictive value; NPV, negative predictive value; PLR, positive likelihood ratio; NLR, negative likelihood ratio.

^*^The optimal cutoff was determined by the receiver operating characteristic curve and the maximum Youden index (sensitivity + specificity - 1) in the training set.
