# Supplementary material for: Major Adverse Kidney Events in Hospitalized Older Patients With Acute Kidney Injury: Machine Learning–Based Model Development and Validation Study
Source: J Med Internet Res. 2025 Jan 3;27:e52786. doi: 10.2196/52786 (PMC11748444; doi:10.2196/52786)

SHAP dependence plots of the model. SHAP, SHapley Additive exPlanations; RDW-CV, red blood cell distribution width-coefficient of variation.


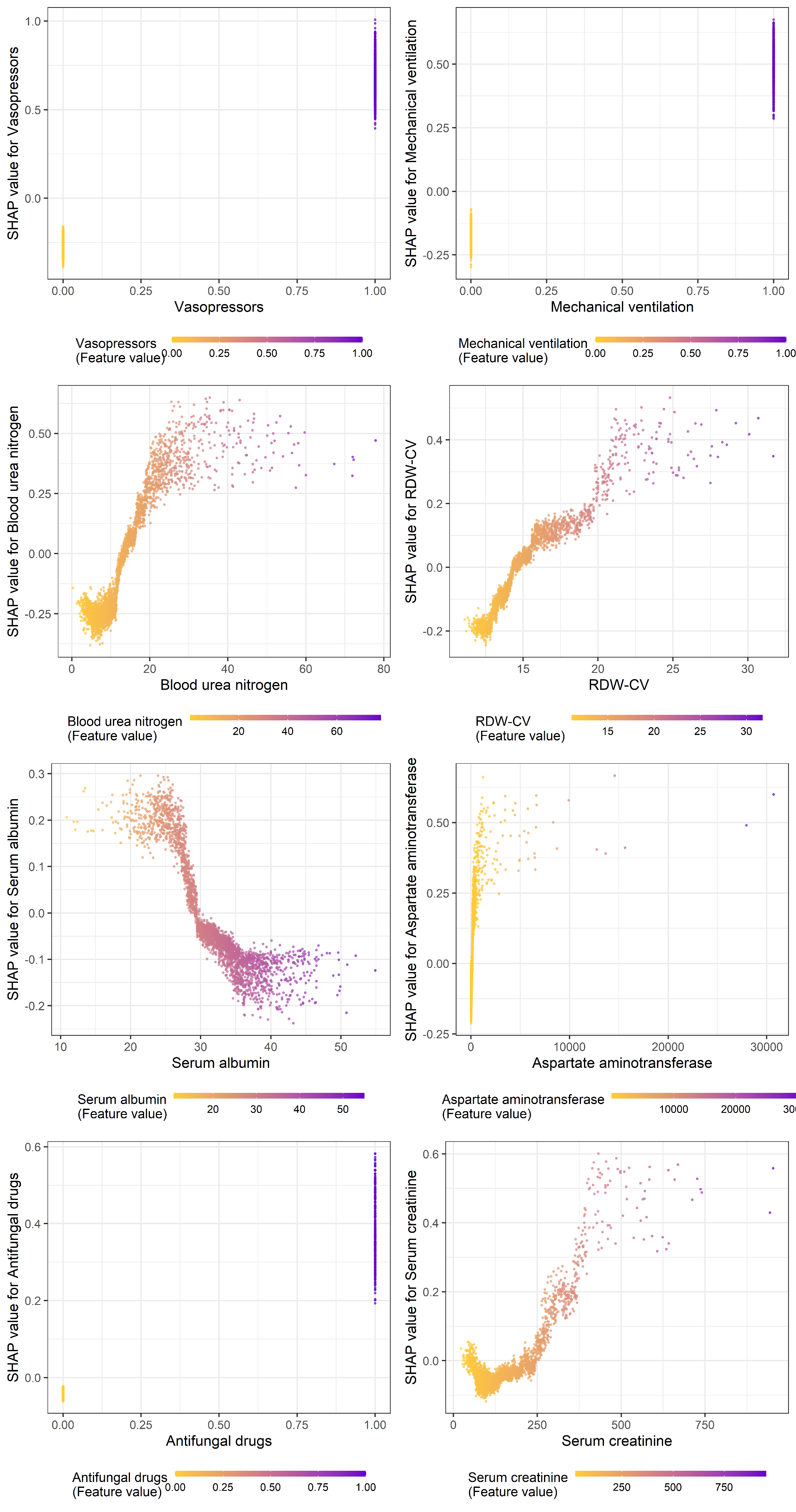

Supplement: Multimedia Appendix 9 [file jmir_v27i1e52786_app9.docx]
