# Supplementary material for: Major Adverse Kidney Events in Hospitalized Older Patients With Acute Kidney Injury: Machine Learning–Based Model Development and Validation Study
Source: J Med Internet Res. 2025 Jan 3;27:e52786. doi: 10.2196/52786 (PMC11748444; doi:10.2196/52786)

Receiver operating characteristic curves of the model in distinct age groups within the training set (A) and internal test set (B). AUC, area under the curve.


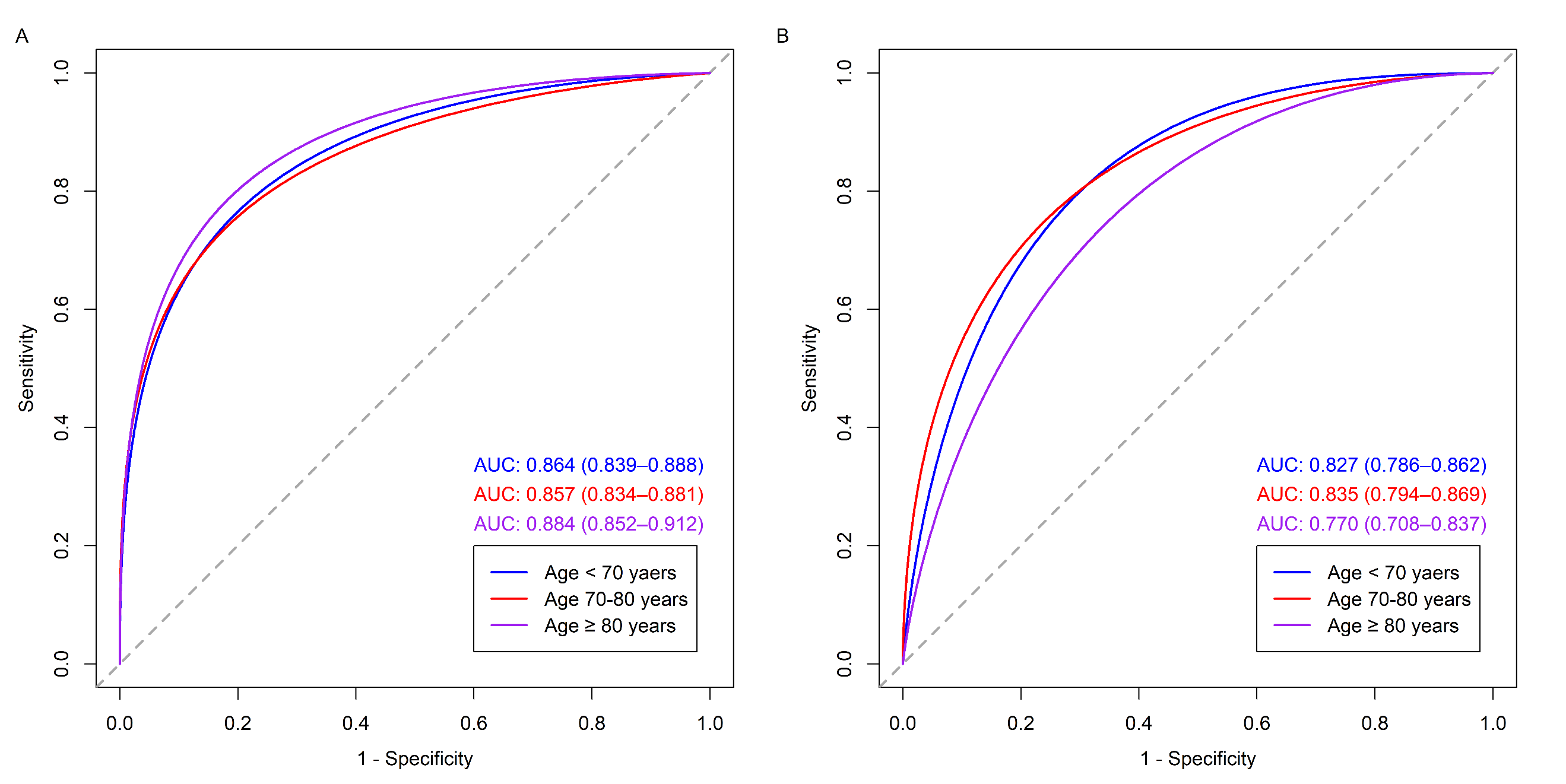


Receiver operating characteristic curves of the simplified model in distinct age groups within the training set (A), internal test set (B), and external test set (C). AUC, area under the curve.


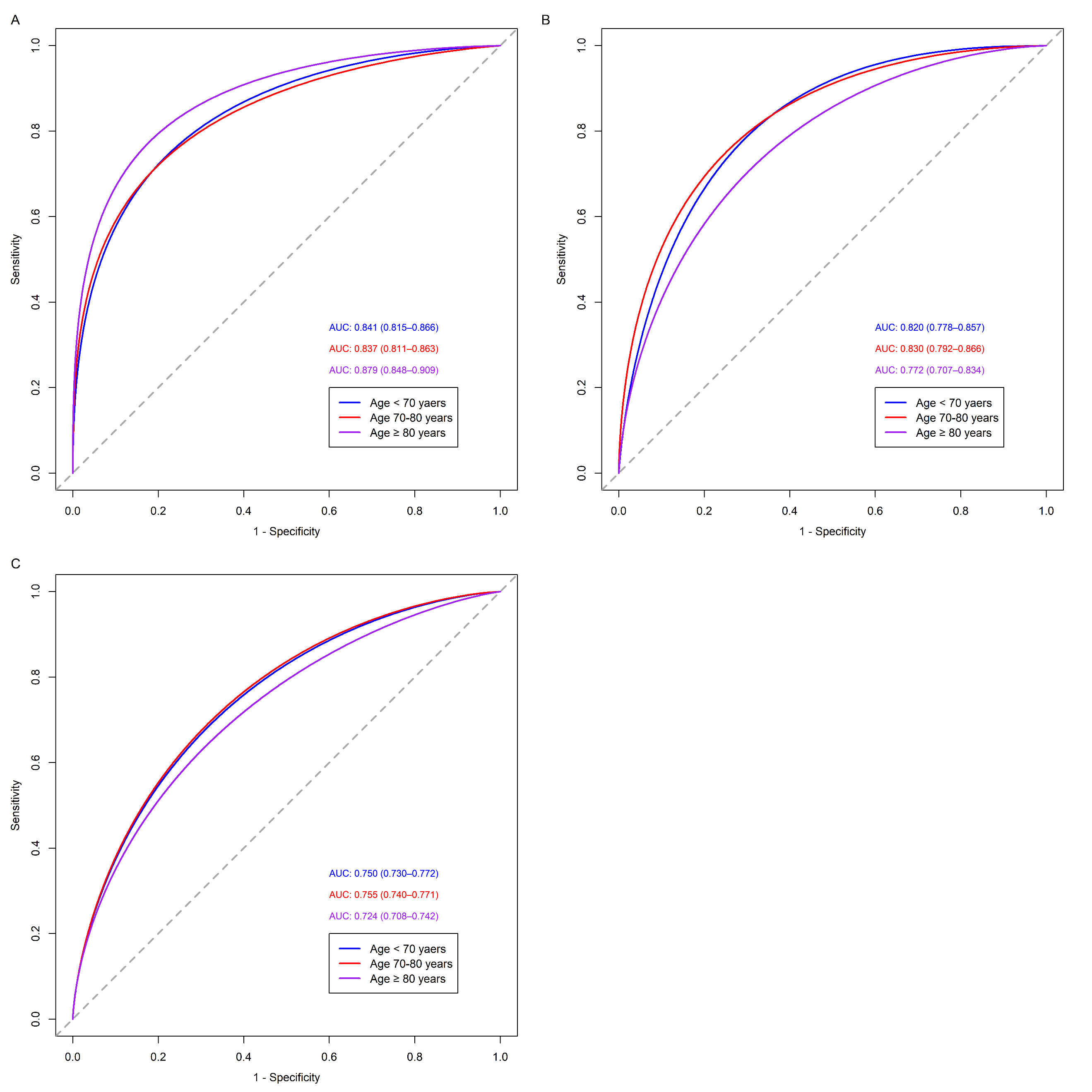

Supplement: Multimedia Appendix 10 [file jmir_v27i1e52786_app10.docx]
